# Re-Thinking Subjective Wellbeing of Latin-American and Caribbean Men With Epilepsy: Beyond Sexual Dysfunctions Issues

**DOI:** 10.3389/ijph.2023.1605280

**Published:** 2023-02-10

**Authors:** Pedro Paulo Gomes do Nascimento, Luciana Patrizia Alves de Andrade-Valença

**Affiliations:** Neurology Unit, Hospital das Clínicas, Medical School of Recife, Federal University of Pernambuco, Recife, Brazil

**Keywords:** quality of life, burden of disease, masculinity, sexual and reproductive healthcare, positive mental health

**The IJPH series “Young Researcher Editorial” is a training project of the Swiss School of Public Health.**

Epilepsy is a common neurological disease that affects the quality of life of approximately 6.3 million people in Latin America and the Caribbean (LAC) [1]. It is characterized by an enduring tendency to experience epileptic seizures and the neurobiological, cognitive, and psychosocial consequences of these seizures. Epilepsy affects people of all ages, with a similar prevalence among men and women living in LAC (410 vs. 413 per 100,000 population, respectively).

In 2019, a total of 1,375,066.9 disability-adjusted life years (DALYs) in LAC countries were attributed to epilepsy, with men being more affected. Three potential reasons may explain higher DALYs in men with epilepsy (MWE): 1) steroid hormones—adrenal corticosteroids and androgenic steroids—may increase seizure sensibility; however their molecular mechanisms are unclear 2) more epilepsy cases associated with exposure to potential injury triggers such as alcohol consumption, occupational or traffic accidents, and 3) higher incidence of Sudden Unexpected Death in Epilepsy (SUDEP) in males, particularly in the context of alcohol use [1].

Depression and anxiety are important comorbidities associated with epilepsy [2]. These mental health issues can significantly impact the overall wellbeing of people with epilepsy and affect both genders. Mental disease occurrence is exacerbated by social factors such as stigma and discrimination, especially for MWE. In addition, their diagnosis and treatment are often neglected.

In general, health promotion interventions and policies that aim to promote gender equity tend to focus on improving the health of children and women, while men’s health issues are stigmatized and tend to be ignored [3]. This tendency is also observed in epileptology, the study and treatment of epilepsy. Despite MWE experiencing gender-specific issues cited above, data on MWE tends to ignore the influence of psychosocial factors, focusing instead on the occurrence of sexual dysfunction (SD) [4]. In fact, MWE report sexual problems more often than healthy men. The higher occurrence of SD among MWE may be due to epilepsy, the use of anti-seizure drugs, and mental health issues. However, MWE quality of life scores are influenced more by depression and anxiety occurrence than clinical issues such as seizure control or SD [4].

A better understanding of the inter-relation between mental health, wellbeing, and social factors for MWE health is necessary, but data about this question are scarce. The influence of sociocultural aspects on the behavior of chronically ill men has been evaluated in other clinical contexts, such as prostate cancer or psychiatric disorders. Evidence suggests that hegemonic social constructions of masculinity—including heterosexuality, assertiveness, self-control, physical strength, and emotional restraint—define an ideal man [5]. Chronically ill men who cannot attain the ideal of masculinity may experience subordination and marginalization. This can lead to low self-esteem, an increased burden of chronic diseases, and a decreased sense of wellbeing. This may in turn contribute to reduced sexual satisfaction, and increase the likelihood of suffering from mental disorders [5].

To understand these relationships, it is necessary to define subjective wellbeing (SWB). SWB is a personal feeling of life satisfaction that is comprised of three components: 1) the presence of emotional positive aspects (e.g., happiness, pleasure); 2) the absence of negative emotional effects (e.g., depressed mood and stress), and 3) cognitive wellbeing (satisfaction or dissatisfaction from life events, as from one’s job or marriage) [6]. High SWB levels result from balance on positive and negative life events impact, which are influenced by individual psychological skills [6]. Similarly, sexual satisfaction can be influenced both by positive factors (relationship satisfaction, self-esteem, pleasure) and negative factors (such as threats of failure and its consequences). As a result, sexual wellbeing is derived from positive sexual experiences, negatively affected by SD and modulated by cognitive mechanisms [7]. Thus, it is crucial to recognize MWE as a marginalized population and conduct studies on their SWB, sexual satisfaction, and psychological wellbeing, especially in the LAC context. Their results will aid policymakers in developing better policies and promote effective interventions.

Increasing MWE wellbeing in LAC is a challenge, and strategies based on Positive Psychological Interventions (PPI) may offer a solution. PPI is a branch of psychology that aims to develop interventions such as the identification of character strengths, and exercises for forgiveness or gratitude, to promote wellbeing—including people with epilepsy [8, 9]. PPIs are effective for the promotion of psychological wellbeing and represent an alternative to treating populations with depression, anxiety, and stress. For men, these interventions promote self-kindness and resilience, skills that can foster positive masculinity [7]. As with men with other disabilities, positive male conceptions in MWE could help improve wellbeing, mental health and increase sexual satisfaction [10].

In conclusion, besides improving access to healthcare that includes adequate diagnosis and treatment for epilepsy and mental health comorbidities, such as PPI, it is necessary to implement policies that recognize the importance of psychosocial factors in improving subjective wellbeing for MWE. This will only be possible by better understanding the mental health and related social factors of MWE through research.
